# Preoperative planar lymphoscintigraphy allows for sentinel lymph node detection in 51 dogs improving staging accuracy: Feasibility and pitfalls

**DOI:** 10.1111/vru.12995

**Published:** 2021-06-15

**Authors:** Martina Manfredi, Donatella De Zani, Lavinia Elena Chiti, Roberta Ferrari, Damiano Stefanello, Chiara Giudice, Vincenzina Pettinato, Maurizio Longo, Mauro Di Giancamillo, Davide Danilo Zani

**Affiliations:** ^1^ Department of Veterinary Medicine Università degli Studi di Milano Lodi Italy; ^2^ Centro Clinico‐Veterinario e Zootecnico‐Sperimentale, Università degli Studi di Milano Lodi Italy; ^3^ Fondazione IRCCS Ca’ Granda Ospedale Maggiore Policlinico Università degli Studi di Milano Milano Italy

**Keywords:** 99mTc, lymphatic metastasis, lymphoscintigraphy, sentinel lymph node, tumor staging

## Abstract

Sentinel lymph node (SLN) mapping is the current gold standard for the oncological staging of solid malignancies in humans. This prospective observational study describes the feasibility and the limits of preoperative lymphoscintigraphy for SLN detection in dogs with spontaneous malignancies and the improvements in staging accuracy. Client‐owned dogs with confirmed malignant neoplasia and absence of distant metastasis were prospectively enrolled. Lymphoscintigraphy was performed after the peritumoral injection of Technetium‐99m labeled nanocolloids. Regional dynamic and static images were acquired, with and without masking of the injection site with a lead shield. The dogs were then subjected to surgery for tumor excision and SLN extirpation. Intraoperative SLN detection was performed by combining methylene blue dye and a dedicated gamma probe. Overall, 51 dogs with a total of 60 solid malignant tumors were enrolled. Lymphoscintigraphy identified at least one SLN in 57 of 60 cases (95%). The SLN did not always correspond to the regional lymph node (35/57, 61.4%). The use of a lead shield, masking the injection site, markedly improved the SLN visibility. The median time of SLN appearance was 11.4 ± 9.3 min. No side effects were observed. Preoperative lymphoscintigraphy allows for SLN detection in dogs and can improve staging accuracy by either identifying the SLN in a different lymphosome than clinically expected or discriminating the draining node in uncertain cases. The combined use of preoperative and intraoperative techniques is recommended to increase the SLN detection rate.

**Abbreviations**
99m−Tc: Technetium‐99 m
LN: lymph node
MB: methylene blue
RLN: regional lymph node
SLN: sentinel lymph node

## 1 INTRODUCTION

The sentinel lymph node (SLN) is the first lymph node (LN) that receives direct lymphatic drainage from a primary tumor site.^1^ In veterinary medicine, sampling the regional lymph nodes (RLN), only when palpably enlarged, is notoriously inaccurate, whereas sampling an inappropriate LN might lead to false‐negative results and thereby understaging.^2^, ^3^, ^4^ Tumor‐induced lymphangiogenesis increases the variability of lymphatic drainage patterns, thus hampering the possibility of SLN identification based on anatomical location alone.^5^ Therefore, SLN mapping procedure indicates the correct LN to be assessed for the presence of nodal metastasis, allowing a more accurate tumor staging, thus aiding treatment decision and prognostication.^6^, ^7^, ^8^, ^9^ Furthermore, a potential therapeutic role of early nodal intervention in oncological patients with no signs of lymphadenopathy has been hypothesized, although it remains to be investigated in both, human^10^ and canine^11^ oncology.

Different mapping techniques have been developed in human and veterinary oncology based on the same principle i.e., to inject a tracer at the primary tumor site and follow the lymphatic pathways to the first draining LN.^12^, ^13^, ^14^, ^15^, ^16^ Methylene blue (MB) was the first tracer used for lymphadenography in patients with penile carcinoma.^17^ Radiocolloids, associated with blue dyes, were first used in human oncology for SLN mapping in patients with melanoma^18^ and, among other malignancies, the breast^19^ and prostate^20^ cancer. Radiocolloids for SLN mapping includes preoperative planar lymphoscintigraphy as well as intraoperative nodal detection with a hand‐held gamma probe. The most common radionuclide is the Technetium‐99m (99m−Tc), which has a physical half‐life of 6.01 h and emits gamma (photons) rays between 140.5 keV (98.6%) and 142.6 keV (1.4%).^21^ Colloids used for radionuclide labeling includes the sulfur colloid (<200 nm), human serum albumin nanoparticles (<80 nm), and antimony sulfide colloid (10 nm). Although tracers with smaller particle sizes ensure visualization of a greater number of LNs, they lead to difficulties in image interpretation and intraoperative SLN identification.^12^

Only a few studies have described the use of lymphoscintigraphy for SLN mapping in veterinary patients with spontaneous tumors,^3^, ^6^, ^8^, ^14^ likely due to the restrictive access to radioactive tracers and the high cost of the equipment. Nevertheless, lymphoscintigraphy is still the gold standard in human oncology^22^ for SLN mapping in patients affected by cutaneous melanoma^23^ and breast cancer,^24^ as it allows for the identification of both the internal and superficial SLNs and aid in preoperative surgical planning.

The present study aimed to describe the feasibility and effectiveness of preoperative lymphoscintigraphy for SLN detection in canine patients with spontaneous malignancies in different anatomical locations. We further investigated whether the first LN draining the tumor site could be identified within unexpected lymphatic basins compared to anatomic RLNs.

We hypothesize the following: (a) peritumoral injection of ^99^ ^m−^Tc‐labeled nanosized human serum albumin allows rapid detection of SLN and the associated lymphatic pathway; (b) the first LN draining the tumor site can be identified within unexpected lymphatic basins compared to anatomic RLNs.

## 2 MATERIALS AND METHODS

### 2.1 Patients

In this prospective observational study, a convenience sample without a predetermined cohort size was enrolled. Client‐owned dogs admitted to a veterinary institution for oncological surgery between June 2016 and October 2019 and meeting the inclusion criteria were included. A part of the patients (30 dogs) was included in a study concerning the impact of SLN biopsy on oncological staging in dogs with mast cell tumor.^6^ The inclusion criteria were as follows: (a) cytological or histologically confirmed spontaneous malignant neoplasia (first presentation or recurrence), (b) tumors amenable for surgical excision and accessible for peritumoral injection; (c) absence of thoracic, abdominal, and/or musculoskeletal metastasis at admission, according to the staging reference guidelines for each tumor (ultrasound‐guided cytology of spleen and liver for mast cell tumors, contrast‐enhanced computed tomography of the thorax and abdomen for epithelial and mesenchymal neoplasia). We excluded dogs with clinically abnormal RLN and cytological evidence of metastases (nodal macrometastases). The final decision for subject's inclusion was made by two veterinarians with, respectively, more than 10 (R.F.) and 15 years (D.S.) of documented experience in surgical oncology. All the procedures were conducted following the European legislation concerning Animal Protection and Welfare (Directive 2010/63/EU) and the Italian ethical law (Decreto Legislativo 04/03/2014, n.26). A client‐signed informed consent was obtained for each procedure. Since the data used in this study were collected as part of a routine clinical activity, no ethical committee approval was required. Each animal received appropriate tumor staging, blood testing, and presurgical workup according to the type of neoplasia, comorbidities, and/or presenting clinical signs.

### 2.2 Planar lymphoscintigraphy

Lymphoscintigraphy was performed with animals under general anesthesia. A 99m−Tc labeled nanosized human serum albumin (NANOALBUMON, Radiopharmacy Laboratory Ltd., Budaörs, Hungary) solution was prepared using 0.9% NaCl as a solvent. Each patient received 10–35 MBq/0.5 ml, injected using a 25‐G needle subcutaneously in four quadrants around the primary tumor. The MBq dose of the syringe was measured using a dose calibrator (ATOMLAB 100Plus, Biodex Medical Systems, Shirley, New York), before and after the injection to calculate the administered dose.^3^

Regional dynamic images (ventral view, 1 frame/s for 60 s, matrix size 256 × 256, energy window 140 KeV ± 10%) were captured immediately after the injection (T0), then after 3 (T1) and 8 (T2) min with a single head gamma camera (Picker Prism 2000XP, Picker International, Highland Heights, Ohio) with an low energy high resolution parallel hole collimator.

**FIGURE 1:**
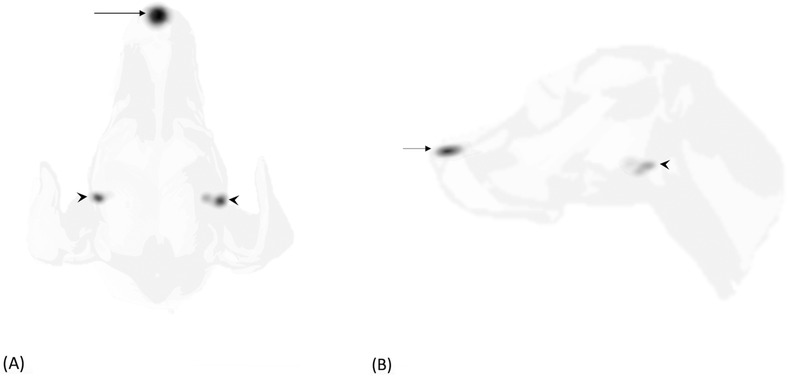
Dorsal (A) and right lateral (B) planar static images (120 s/frame, matrix size 256 × 256) of the head of a dog with a single MCT at the planum nasale, acquired 3 minutes after injection. Injection site (arrow) was not masked and a bilateral radiopharmaceutical uptake was observed at the level of mandibular LNs (arrowheads)

**FIGURE 2:**
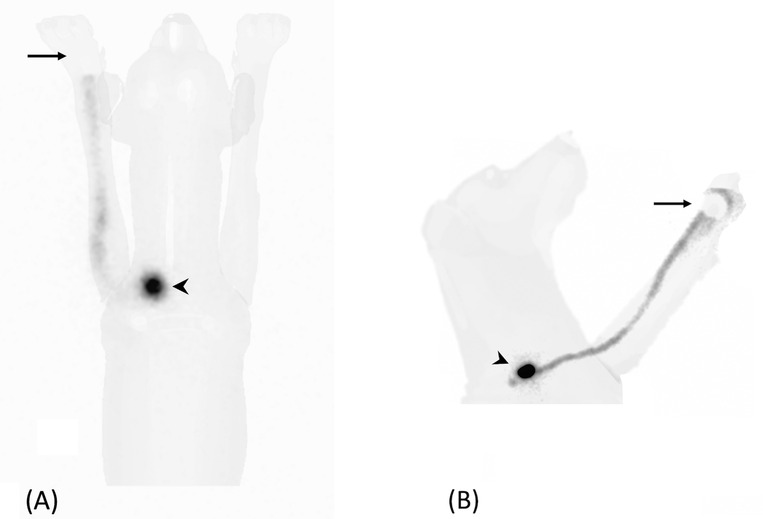
Ventral (A) and left lateral (B) planar static images (120 s/frame, matrix size 256 × 256) of a dog with a digital MCT in the left hand. Injection site (arrow) was masked with a 2 mm thick lead shield. A focal radiopharmaceutical uptake is visible at the level of the left superficial cervical LN (arrowheads)

Two ventral or dorsal images and two lateral static images (Figures 1A,B and 2A,B), centered on the tumor site, were acquired (120 s/frame, matrix size 256 × 256) immediately after the dynamic study. Each lateral (left or right) and ventral or dorsal views were selected based on the minor distance between the tumor and the gamma camera to improve the lymphatic uptake visualization. For one image per positioning, the injection site (IS) was masked with a 2 mm thick lead shield of appropriate size. If required, additional images were taken by centering the gamma camera cranially/caudally to the region of interest or by changing the patient's positioning until the first draining LN was identified as a distinct radiopharmaceutical uptake.

Looking at the real‐time images of the gamma camera persistence oscilloscope (P‐scope), the syringe with residual dose was used as a pointer to identify the SLN based on the syringe position relative to the patient's anatomical structures.^3^, ^23^ All the procedures were performed by two veterinarians with, respectively, more than 10 (D.D.Z.) and 15 years (D.Z.) of documented experience in veterinary radiology and nuclear medicine. The SLN identification was reached by consensus. In patients with more than one primary tumor, the injection and image acquisition were performed separately for each tumor in the same session.

The occurrence of adverse events after the radiopharmaceutical injection was evaluated and recorded by the veterinary surgeon and the veterinary radiologist in charge of the case. The primary tumor area was checked prior, during and at the end of the scintigraphic procedure. The clinical record had reported any macroscopically visible alteration. For systemic side effect, during the general anesthesia, the cardiovascular and pulmonary parameters were checked and reported on the anesthesiology record, any unpredictable changes had been described.

### 2.3 Intraoperative SLN detection

After planar lymphoscintigraphy, the animals were admitted to surgery for tumor excision and SLN extirpation *lege artis*. Prior to the surgical site's aseptic preparation, 0.4 ml of 5 mg/ml sterile MB (S.A.L.F. S.p.A, Cenate Sotto, Bergamo, Italy) was peritumorally injected in the radiopharmaceutical inoculation sites.^3^, ^6^, ^8^, ^14^ The audible sounds and counts visible on the gamma probe console (Crystal probe SG04, Crystal Photonic GmbH, Berlin, Germany), alongside MB visualization, guided the surgical dissection during the lymphadenectomy procedure. Any LN presenting a blue stain or a radioactive count (RC) at least twice the RC of a distant body region (background counts), was extirpated. Non‐hot and non‐blue nodes within the sentinel lymphocentrum were extirpated if visible without further surgical dissection, in order to reduce the risk of residual disease. The surgeon then checked the operation site with the gamma probe and evaluated the residual radioactivity. Any other LN with an RC equal to or greater than 10% of the LN with the highest RC,^6^, ^8^ was removed. Primary tumor and extirpated LN were checked ex vivo with the detector and then fixed in 10% neutral‐buffered formalin for a routine histopathological examination.^7^ Animals were hospitalized for at least 24 h after surgery.

### 2.4 Data analysis

Descriptive analysis was performed by a veterinarian with coursework training in statistics (MM). The following data were organized in a spreadsheet (Microsoft Excel for Mac 2019 Version 17.0): patient signalment, tumor type, presentation (first vs recurrence), and the localization based on the lymphatic territories identified by Suami and colleagues.^26^ Sentinel lymph nodes detected with lymphoscintigraphy, gamma probe, and MB were recorded and compared to the RLNs reported to normally drain the tumor's anatomical territory.^26^ Other recorded data included the effect of the IS masking on SLN visibility during lymphoscintigraphy, the presence or absence of histopathologic LN metastasis, radiotracer immediate and delayed side effects.

## 3 RESULTS

### 3.1 Patients

Fifty‐one dogs were enrolled in the study. The ages of dogs ranged from 1 to 15 years (mean, 7.9 ± 3.2 years; mode, 10 years; median, 8 years); weights ranged from 3.2 to 51 kg (mean, 26.8 ± 12.4). Twenty‐eight of the dogs were male (10 neutered) and 23 were female (20 spayed). There were 12 mixed breed dogs; nine Labrador Retrievers; six Golden Retrievers; three Dogo Argentinos; two Boxers; two American Staffordshire Terriers; two English setters, and one each of other 15 breeds. The total number of tumors investigated in the dogs were 59, including mast cell tumor (MCT, n = 47), mammary adenocarcinoma (n = 3), oral melanoma (n = 2), thyroid carcinoma (n = 2), sub‐lingual squamous cell carcinoma (SCC, n = 1), parotid adenocarcinoma (n = 1), perivascular wall tumor (myopericytoma, n = 1), oral undifferentiated sarcoma (n = 1), and benign mixed mammary tumor (n = 1). The inclusion of benign mammary neoplasia was due to an initial diagnosis of a malignant tumor on the cytological examination. The patient presented again after 2 months for an SLN mapping of adenocarcinoma on the contralateral mammary chain. Another dog with multiple cutaneous MCTs, presented after 2 years for MCT located in a different lymphatic territory. Six dogs had more than one primary tumor: two presented with three cutaneous MCTs (n. 6, 31), three had two cutaneous MCTs (n. 10, 13, and 17), and one had two mammary adenocarcinomas (n. 41). Two dogs presented with MCT recurrences; in particular patient n. 3 underwent MCT excision in association with a right superficial cervical lymphadenectomy and adjuvant radiation therapy. The distribution of lymphatic territory based on the tumor site is shown in Supporting Information 1.

### 3.2 Sentinel lymph node detection

Lymphoscintigraphy located the SLN in 57 of the 60 tumors (95%). Dynamic images allowed SLN identification in 15 of 57 cases (T0 in 14 cases, T2 in 1 case), whereas only a lymphatic radiotracer pathway was visible in the other 23 procedures.

Subsequent static images detected SLN in all 57 cases; in 22 of 57 tumors, the SLN was observed on the first static image (9 or 10 min after injection). The median time of SLN appearance on planar images was 11.4 ± 9.3 min (range: 9‐42 min). The use of a 2 mm lead shield covering the IS subjectively improved SLN visibility during the lymphoscintigraphic procedure in 41 of 57 cases (72%).

The radiotracer failed to identify the SLN in three cases. In the first dog with palpable thyroid carcinoma, a local radiotracer diffusion was observed after the peritumoral subfascial injection, but neither draining node nor lymphatic pathways were detected. Similarly, for patient n. 3, which presented an MCT recurrence after surgical excision, only a subcutaneous radiotracer diffusion was observed without SLN identification. In the other dog with a thyroid tumor, an ultrasound‐guided intratumoral radiotracer injection was performed,^27^ but no radiocolloid migration was observed.

The LNs identified with lymphoscintigraphy corresponded to RLN in 22 of 57 cases (38.6%). In 10 of the 22 matching cases, lymphoscintigraphy allowed for SLN identification within the axillary lymphatic territory among the axillary LN, accessory axillary LN, or both. In 18 cases (31,6%), the tumor anatomical site was located between two different lymphosomes and lymphoscintigraphy allowed the discrimination of the draining SLN. In eight cases (14%), lymphoscintigraphy detected a second SLN in a different lymphatic territory from the tumor lymphosome. In nine cases (15.8%), the identified SLNs did not correspond to the RLN.

The gamma probe intraoperatively detected the radioactive SLNs within the lymphatic basin identified by lymphoscintigraphy in all the cases. In two patients (n. 16, 28) with an MCT of the right stifle, the gamma probe identified an additional radioactive LN in the popliteal basin, which was not recognized by lymphoscintigraphy. Methylene blue injection was used in 51 cases and identified the draining LNs in 46 (90.2%). Blue‐stained LNs corresponded to radioactive LNs in 45 cases. In a dog with sub‐lingual SCC (n. 33), MB identified an additional metastatic LN in the contralateral mandibular basin. In patient number 3, where the radiotracer failed to identify the SLN, the MB migrated to the LN draining the MCT recurrence site. In one case (n. 44) with a sternal MCT, MB stained the left superficial cervical LN but not the contralateral radioactive LN; both were confirmed as early metastatic on the histopathological examination (HN2^6^). In four cases, the MB did not stain any LN.

Patient n. 22 (MCT of the right stifle) showed a radiotracer and MB uptake of a right inguinal structure, that was histologically identified as focal inflammatory infiltration of connective tissue composed by mast cells and eosinophils. This patient had previously undergone an ipsilateral unilateral mastectomy for a mammary tumor.

Lymphadenectomy was performed in 53 of 60 tumors. As for the MCT cases, histopathological classification^7^ revealed absence of metastasis (HN0) in 15 of 44 cases, premetastatic nodal lesion (HN1) in seven of 44 cases, early metastasis (HN2) in 18 of 44 cases, and overt metastasis (HN3) in four of 44 cases. As for the other malignancies, two of nine patients presented metastasis on the histopathological examination (n. 33, SCC; n. 38, oral melanoma).

### 3.3 Side effects

Twelve dogs with MCT showed mild swelling at the site of radiopharmaceutical injection. Duration of swelling was not evaluated due to the immediate admission of the patients to surgical extirpation of tumor. Both dogs with oral melanoma and the dog with sub‐lingual SCC showed minimal bleeding related to the injection procedures, which was resolved immediately with direct pressure hemostasis.

## 4 DISCUSSION

This prospective study demonstrates the feasibility, safety, and effectiveness of preoperative lymphoscintigraphy for SLN mapping in dogs with malignant tumors. Peritumoral injection of 99m−Tc‐labeled nanocolloids allowed for rapid detection of the SLN and the associated lymphatic pathway in 57 of 60 cases, without any relevant injection‐induced local reactions or adverse side effects. The mild swelling observed at the injection site in 12 dogs could be related to induced MCT degranulation as well as a reaction to the administered radiopharmaceutical. The bleeding observed in dogs with oral cavity tumor was most probably related to the injection procedure.

Randall et al^14^ recently reported a 100% SLN detection rate with lymphoscintigraphy in 20 dogs with head and neck malignancies. In our study population, lymphoscintigraphy failed to identify SLN in three dogs, two of which had thyroid carcinoma. Successful detection of SLN in dogs with thyroid neoplasia has been previously described.^3^ In our investigation, one dog had a palpable thyroid carcinoma and received a peritumoral radiotracer injection that showed local activity diffusion without any lymphatic vessels or node detection. The second patient received an intratumoral ultrasound‐guided radiotracer injection, as described in human oncology,^27^ but no lymphatic drainage could be detected within 1 h of the injection. In human patients with thyroid tumors, lymphoscintigraphy is performed up to 2 h after the radionuclide injection^27^; therefore, a possible source of error may have been the short timing between the radiotracer injection and image acquisition. In the present study, lymphoscintigraphy was performed immediately before surgery and the dog was moved in the operating theater to avoid prolonging the duration of general anesthesia. Further studies are warranted to assess whether the migration of the radiotracer requires a longer time in thyroid tumors and to define a specific thyroid SLN mapping protocol for small animals. The third patient in which lymphoscintigraphy failed to identify the SLN, presented with an MCT recurrence. The patient also underwent superficial cervical lymphadenectomy during the first surgical session and adjuvant radiation therapy for nodal metastasis. In humans, the impact of scar tissue on SLN detection is controversial. Few studies have reported decreased SLN identification rate in patients where the lymphatic network was disrupted by a previous surgery^28^, ^29^ or radiotherapy.^30^ Hlusko et al^31^ investigated the impact of surgery on the drainage patterns from the canine brachium, demonstrating a partial agreement between preoperative and postoperative lymphoscintigraphy, and Vasques et al^32^ showed that para‐areolar incision in the upper outer quadrant of the first mammary gland did not significantly interfere with SLN identification in a canine model. However, both studies were conducted on healthy dogs, and data on tumor‐bearing dogs is still lacking. Possible factors influencing the SLN detection rate in these cases are the interval between surgery and the successive SLN mapping procedure, formerly resected tissue volume and location, the tracer IS, and previous radiotherapy. Nonetheless, in human oncology the affirmed ASCO guidelines still recommend SLN biopsy for this group of patients.^33^ For the dog in our study, the SLN could be identified with an MB peritumoral injection. Radiotracer failure could be attributed to an error in the radiopharmaceutical preparation, in the image analysis, or due to the different tracer absorption levels. However, the presence of an inguinal radioactive blue‐stained connective tissue structure in another dog, that had previously undergone mastectomy (n. 22), further confirmed the abnormal tracer uptake in the presence of disrupted lymphatic drainage due to an old surgical scar.

Early dynamic images in the preoperative lymphoscintigraphy protocol did not offer additional clinical information on SLN detection, compared to static images, which is in line with previous research in human literature.^34^ After the first appearance of the SLN on the dynamic or static images, orthogonal views need to be combined to correctly identify the SLN basin. To overcome the lack of anatomical details provided by lymphoscintigraphy, a syringe with a residual radiopharmaceutical was used as a pointer, as previously described.^3^, ^25^ Under the guidance of real‐time images displayed on the gamma camera P‐scope, the position of the pointer in relation to patient anatomical landmarks allowed the identification of the SLN basin. Although not used in this study, cobalt body markers or systemic administration of 99m−Tc could further improve SLN identification, providing the visualization of body contour.^25^

The differences observed in the SLN identification timing could be attributed to individual variations in lymphatic flow velocity as well as the animal's position and the subsequent effect of the bodyweight on tissues.^14^, ^15^ This was not investigated in the current study and requires further research. The close proximity between the IS and the SLN basin can also lead to a challenging visualization of the LN uptake (“shine‐through effect”^35^). The use of a 2 mm lead shield to cover the IS subjectively improved the SLN visibility during the procedure and reduced the concealing of the highly radioactive site on nearby nodes.

However, in two dogs (n. 16 and 28) with an MCT of the right stifle, the intraoperative gamma probe identified an additional radioactive LN in the popliteal basin, which was not identified with lymphoscintigraphy, probably due to superimposition between the IS and the LN. In this study, the intraoperative use of a gamma probe allowed a targeted identification of radioactive SLN included in the lymphatic basin detected by preoperative planar lymphoscintigraphy.

In the dog presenting with sub‐lingual SCC (patient n. 33), MB identified an additional LN in the contralateral mandibular basin, which was not radioactive but was metastatic. In human oncology, the failure of SLN identification is associated with an increased risk of metastases to the axillary lymph system.^29^ Recently, Rossi et al^36^ described the failure of LN contrast uptake, using computed tomography lymphography, in dogs with nodal macrometastasis. These findings highlight the importance of case selection among patients with clinically negative RLN and the combination of two mapping techniques (radiopharmaceutical and blue dye injection) to improve the SLN detection rate. In the authors’ opinion intraoperative and preoperative techniques serve complementary purposes: preoperative planar lymphoscintigraphy provides the indication of which lymphocentrum needs to be surgically explored, while intraoperative techniques guide the surgical dissection.

The results confirm the hypothesis that the first LN draining the tumor site could be identified within unexpected lymphatic basins. As previously stated,^6^, ^8^, ^9^ SLN mapping is a keystone concept in canine oncological staging, identifying the correct LN to sample in order to improve patient prognostication and adjuvant therapy selection.

An important limitation of scintigraphy is the need for authorization and equipment for radioactive isotope use, which limits the number of facilities with nuclear medicine services. In addition, specific precautionary procedures are required to guarantee personnel safety, despite radiation exposure being reported to be minimal.^8^ As a consequence, other SLN mapping techniques have been developed in human medicine and further described for oncological staging purposes in dogs, such as the radiographic lymphography,^35^ CT lymphography,^14^, ^15^, ^36^, ^37^, ^38^ contrast‐enhanced ultrasound,^16^, ^39^ and near‐infrared imaging.^40^, ^41^

In this study, we decided to perform SLN extirpation and histopathological assessment without prior nodal sampling. This was motivated by the reported low sensitivity and specificity of fine needle cytology compared to histopathology^42^ in assessing LN metastatic status, and, in dogs with MCT, by the importance of histological lymph node categorization^7^ and the hypothesized therapeutic role of lymphadenectomy.^11^, ^43^ The long‐term effects of removing normal LN is still unknown and worthy of further investigations.

In conclusion, preoperative planar lymphoscintigraphy is a feasible, safe, and effective method for SLN detection in dogs with spontaneous malignancies in different anatomical locations. This mapping technique drives the surgical extirpation of SLNs in a specific lymphocentrum, allowing for accurate oncologic staging, either for identifying the tumor‐draining node within unexpected lymphatic basins or discriminating the draining node in uncertain cases. The use of combined preoperative and intraoperative techniques is recommended to improve the SLN detection rate.

## LIST OF AUTHOR CONTRIBUTIONS

### Category 1

- Conception and Design: De Zani, Manfredi, Zani, Ferrari, Stefanello, Chiti, Giudice, Longo, Pettinato, Di Giancamillo
- Acquisition of Data: Manfredi, De Zani, Zani, Stefanello, Ferrari, Chiti, Giudice

### Category 2

- Drafting the Article: Manfredi, De Zani
- Revising Article for Intellectual Content: De Zani, Manfredi, Zani, Ferrari, Stefanello, Di Giancamillo, Longo, Chiti, Pettinato, Giudice

### Category 3

(a) Final Approval of the Completed Article: Manfredi, De Zani, Zani, Ferrari, Stefanello, Di Giancamillo, Longo, Chiti, Pettinato, Giudice

## EQUATOR NETWORK GUIDELINES DISCLOSURE

The authors followed AQUA reporting guidelines.

## CONFLICT OF INTEREST

The authors declare no conflict of interest.

## Supporting information


**Supplement 1** Tumor sites, corresponding lymphosomes, and SLN detected with lymphoscintigraphy and intraoperative techniques in 51 dogs. In three patients (n. 10, 18, 43), the intraoperative SLN detection was not performed since the owner refused lymphadenectomy.
