# Supplementary material for: Preoperative planar lymphoscintigraphy allows for sentinel lymph node detection in 51 dogs improving staging accuracy: Feasibility and pitfalls
Source: Vet Radiol Ultrasound. 2021 Jun 15;62(5):602–9. doi: 10.1111/vru.12995 (PMC8518895; doi:10.1111/vru.12995)
Supplement: Supplementary file 1 — Supplement 1 Tumor sites, corresponding lymphosomes, and SLN detected with lymphoscintigraphy and intraoperative techniques in 51 dogs. In three patients (n. 10, 18, 43), the intraoperative SLN detection was not performed since the owner refused lymphadenectomy. [file VRU-62-602-s001.docx]

**Supplement 1** Tumor sites, corresponding lymphosomes, and SLN detected with lymphoscintigraphy and intraoperative techniques in 51 dogs. In three patients (n. 10, 18, 43), the intraoperative SLN detection was not performed since the owner refused lymphadenectomy. Methylene blue injection was not performed in 4 patients (n. 29, 31, 32, 51) and intraoperative gamma probe detection was not possible in 2 cases (n. 44, 46) due to probe malfunctioning.

| **Pt** | **Tumor** | **Tumor site** | **Lymphosome** | **Lymphoscintigraphy** | **Gamma Probe** | **MB dye** |
| --- | --- | --- | --- | --- | --- | --- |
| **1** | MCT | R caudo-ventral neck | R ventral cervical | R sup cervical | R sup cervical | R sup cervical |
| **2** | MCT | R ischiatic region | R hypogastric R inguinal | R inguinal | R inguinal | R inguinal |
| **3** | MCT - recurrence | R shoulder | R dorsal cervical R axillary | none | none | R accessory axillary |
| **4** | MCT | L thorax (XIII rib) | L axillary | L accessory axillary | L accessory axillary | L accessory axillary |
| **5** | MCT | L thorax (XIII rib) | L axillary | L accessory axillary | L accessory axillary | L accessory axillary |
| **6a** | MCT | R stifle | R inguinal | R inguinal | R inguinal | R inguinal |
| **6b** | MCT | L stifle fold | L inguinal  L medial iliac | L inguinal | L inguinal | L inguinal |
| **6c** | MCT | sternum - R lateral | R axillary | R sup cervical | R sup cervical | R sup cervical |
| **7** | MCT | R stifle | R inguinal | R popliteal  R inguinal | R popliteal  R inguinal | R popliteal  R inguinal |
| **8** | MCT | L preputial | L inguinal | L inguinal | L inguinal | L inguinal |
| **9** | MCT | R stifle | R inguinal | R popliteal  R inguinal | R popliteal R inguinal | R popliteal R inguinal |
| **10** | MCT | thorax - R caudodorsal | R axillary | R axillary | no LNA | no LNA |
| **10a** | MCT | thorax - L cranioventral | L axillary | L axillary | no LNA | no LNA |
| **11** | MCT | scrotum | R/L inguinal | R inguinal | R inguinal | R inguinal |
| **12** | MCT | L shoulder | L dorsal cervica L ventral cervical | L sup cervical | L sup cervical | L sup cervical |
| **13a** | MCT | III-IV L mammary gland | L axillary L inguinal | L inguinal | L inguinal | L inguinal |
| **13b** | MCT | III digit R foot | R popliteal | R popliteal | R popliteal | R popliteal |
| **14** | MCT | R forearm | R ventral cervical R axillary | R axillary R sup cervical | R axillary  R sup cervical | R axillary R sup cervical |
| **15** | MCT | thorax - R lateral | R axillary | R axillary | R axillary | R axillary |
| **16** | MCT | R stifle | R inguinal | R inguinal | R inguinal R popliteal | R inguinal  R popliteal |
| **17a** | MCT | L ear base | L dorsal cervical L parotid | L sup cervical | L sup cervical | L sup cervical |
| **17b** | MCT | R suprascapolar region | R dorsal cervical | R sup cervical | R sup cervical | R sup cervical |
| **18** | MCT - recurrence | L stifle fold | L inguinal  L medial iliac | L medial iliac | no LNA | no LNA |
| **19** | MCT | R forearm | R ventral cervical R axillary | R sup cervical | R sup cervical | R sup cervical |
| **20** | MCT | I L mammary gland | L axillary | L axillary L accessory axillary | L axillary  L accessory axillary | L axillary  L accessory axillary |
| **21** | MCT | nasal planum | R/L mandibular | R/L mandibular | R/L mandibular | R/L mandibular |
| **22** | MCT | R stifle | R inguinal | R inguinal * R medial iliac | R inguinal * R medial iliac | R inguinal * |
| **23** | MCT | L thigh - laterocaudal | L inguinal | L inguinal | L inguinal | L inguinal |
| **24** | MCT | tail - R lateral base | R lateral sacral R hypogastric | R medial iliac | R medial iliac | R medial iliac |
| **25** | MCT | preputium - R lateral | R inguinal | R/L inguinal | R/L inguinal | R/L inguinal |
| **26** | MCT | sternum - L lateral | L axillary | L sup cervical | L sup cervical | L sup cervical |
| **27** | Melanoma | mandibular symphysis | R/L mandibular | L mandibular | L mandibular | L mandibular |
| **28** | MCT | R stifle | R inguinal | R inguinal | R inguinal  R popliteal | R inguinal R popliteal |
| **29** | Parotid adenocarcinoma | L parotid gland | L parotid L mandibular | L mandibular L parotid | L mandibular L parotid | NP |
| **30** | MCT | thorax - L lateral | L axillary | L accessory axillary | L accessory axillary | L accessory axillary |
| **31a** | MCT | R thigh - lateral | R inguinal R medial iliac | R inguinal | R inguinal | R inguinal |
| **31b** | MCT | head - L dorsolateral | L dorsal cervical L parotid | L sup cervical | L sup cervical x2 | NP |
| **31c** | MCT | abdomen - L ventrolateral | L axillary | L accessory axillary | L accessory axillary | NP |
| **32** | Thyroid Carcinoma | R thyroid | R mandibular R dorsal superficial R retropharingeal | none | none | NP |
| **33** | SCC | Sub-lingual | R/L mandibular R/L retropharingeal | R mandibular R/L retropharingeal | R mandibular R/L retropharingeal | L mandibular R/L retropharingeal |
| **34** | MCT | L foot | L popliteal | L popliteal | L popliteal | L popliteal |
| **35** | Benign Mammary Tumor | I L mammary gland | L axillary | L accessory axillary | L accessory axillary | L accessory axillary |
| **36** | Thyroid Carcinoma | R thyroid | R mandibular R dorsal superficial R retropharingeal | none | none | none |
| **37** | MCT | L ear base | L dorsal cervical L parotid | L sup cervical | L sup cervical | none |
| **38** | Melanoma | R oral vestibule | R mandibular | R mandibular | R mandibular | R mandibular |
| **39** | Mammary Carcinoma | III-IV L mammary gland | L axillary L inguinal | L inguinal | L inguinal | L inguinal |
| **40** | Sarcoma | L mandible | L mandibular | L mandibular | L mandibular | L mandibular |
| **41a** | Mammary Carcinoma | IV R mammary gland | R inguinal | R inguinal R medial iliac | R inguinal  R medial iliac | R inguinal R medial iliac |
| **41b** | Mammary Carcinoma | V R mammary gland | R inguinal | R inguinal  R medial iliac | R inguinal R medial iliac | R inguinal R medial iliac |
| **42** | MCT | L thigh - medial | L inguinal | L inguinal | L inguinal | L inguinal |
| **43** | PWT | L thigh | L inguinal  L medial iliac | medial iliac | no LNA | no LNA |
| **44** | MCT | first sternebra | R/L axillary R/L ventral cervical | R/L sup cervical | NP | L sup cervical |
| **45** | MCT | L elbow | L axillary | L sup cervical | L sup cervical | none |
| **46** | MCT | L tibia cranio-medial | L inguinal | L inguinal L popliteal | NP | L inguinal L popliteal |
| **47** | MCT | L caudal ear base | L dorsal cervical | L sup cervical | L sup cervical | L sup cervical |
| **48** | MCT | sternum - L cranio lateral | L axillary L ventral cervical | L sup cervical | L sup cervical | none |
| **49** | MCT | sternum - L lateral | L axillary | L accessory axillary | L accessory axillary | L accessory axillary |
| **50** | MCT | R proximal antebrachium | R ventral cervical | R sup cervical  R axillary | R sup cervical  R axillary | R sup cervical  R axillary |
| **51** | MCT | III digit L hand | L ventral cervical | L sup cervical | L sup cervical | NP |

Abbreviations: Pt, patient; MB, methylene blue; MCT, mast cell tumor; SCC, squamous cell carcinoma; R, right; L, left; sup cervical, superficial cervical lymph node; LNA, lymphadenectomy; NP, not performed. *Histopathology identified inflammatory infiltration of connective tissue composed by mast cells and eosinophils.
